# Environmental Fate and Toxicity of Sunscreen-Derived Inorganic Ultraviolet Filters in Aquatic Environments: A Review

**DOI:** 10.3390/nano12040699

**Published:** 2022-02-19

**Authors:** Shengwu Yuan, Jingying Huang, Xia Jiang, Yuxiong Huang, Xiaoshan Zhu, Zhonghua Cai

**Affiliations:** 1 National Engineering Laboratory for Lake Pollution Control and Ecological Restoration, Chinese Research Academy of Environmental Sciences, Beijing 100012, China; dirk_yuan@163.com (S.Y.); jiangxia@craes.org.cn (X.J.); 2 Shenzhen International Graduate School, Tsinghua University, Shenzhen 518055, China; yuan.shengwu@craes.org.cn (J.H.); huang_yuxiong@sz.tsinghua.edu.cn (Y.H.); caizh@sz.tsinghua.edu.cn (Z.C.); 3 School of Environment, Tsinghua University, Beijing 100084, China; 4 Southern Laboratory of Ocean Science and Engineering (Guangdong, Zhuhai), Zhuhai 519000, China

**Keywords:** cosmetics, nanoparticles, environmental behavior, ecosystem, toxic mechanism

## Abstract

An increasing number of inorganic ultraviolet filters (UVFs), such as nanosized zinc oxide (nZnO) and titanium dioxide (nTiO_2_), are formulated in sunscreens because of their broad UV spectrum sunlight protection and because they limit skin damage. However, sunscreen-derived inorganic UVFs are considered to be emerging contaminants; in particular, nZnO and nTiO_2_ UVFs have been shown to undergo absorption and bioaccumulation, release metal ions, and generate reactive oxygen species, which cause negative effects on aquatic organisms. We comprehensively reviewed the current study status of the environmental sources, occurrences, behaviors, and impacts of sunscreen-derived inorganic UVFs in aquatic environments. We find that the associated primary nanoparticle characteristics and coating materials significantly affect the environmental behavior and fate of inorganic UVFs. The consequential ecotoxicological risks and underlying mechanisms are discussed at the individual and trophic transfer levels. Due to their persistence and bioaccumulation, more attention and efforts should be redirected to investigating the sources, fate, and trophic transfer of inorganic UVFs in ecosystems.

## 1. Introduction

Sunscreen is one of the personal care products (PCPs) used to provide protection against ultraviolet radiation (UVR, 10–400 nm) damage [1,2,3]. Recently, with rising production and consumption, sunscreens have been increasingly released into aquatic environments, including oceans, rivers, lakes, and other water bodies, via several means of discharge (e.g., wastewater treatment plant effluents, runoff input, and recreational activities) [4,5,6]. The rapid growth in global tourism, especially coastal and marine tourism, where the number of international tourists worldwide grew from 463 million in 1992 to 763 million in 2004 and is estimated to have reached 1.56 billion in 2020 [7], has contributed to the increasing application of sunscreen [7,8]. Moreover, in these tropical countries, at least 25% of the sunscreens applied to skin are eventually released into the ocean during water recreational activities [9], which could pose potential risks to the aquatic environment.

Sunscreen is a multicomponent product that contains both active ingredients to shield or reflect UVR and commodity coatings to prevent bleaching and the loss of color [10]. The active ultraviolet filters (UVFs) in sunscreens can be organic or inorganic and can reflect and scatter UVR, which protects human skin from direct sunlight radiation [11,12]. Typically, organic UVFs are called chemical filters, as their mode of action (MoA) is related to the chemical changes in their molecules that prevent UVR from reaching the skin. The European Union regulates and authorizes 26 types of organic UVFs (summarized in our previous review) [13], which are widely used and globally recognized. In 2018, the Environmental Working Group (EWG) reported that two-thirds of the 1300 sunscreen products available contain chemicals that the EWG has deemed to be harmful to the environment, which are predominantly organic UVFs [14]. Inorganic UVFs are called physical filters or mineral filters, as their MoA is associated with physical phenomena, such as the scattering and reflection of UVR [15,16,17,18,19,20]. Titanium dioxide (TiO_2_) and zinc oxide (ZnO) are the most widely used inorganic UVFs and are usually present in nanoparticle (NP) form, also known as nanosized TiO_2_ (nTiO_2_) and nanosized ZnO (nZnO), due to their greater dispersion and UV scattering superficial area [14]. Both nTiO_2_ and nZnO are semiconductors with wide band gaps that can effectively shield UV light.

The adverse environmental effects of organic UVFs, including the bleaching effect on coral reefs and the negative hormonal effects on marine animals, were reviewed in a recent study [21]. The ecological risks of organic UVFs have resulted in warnings and restrictions on the application of chemical substances. The Hawaiian state legislature passed a bill on 1 May 2018 that bans the sale and distribution of sunscreens that contain certain organic UVFs (oxybenzone and octinoxate), which is anticipated to become effective in 2021 [22]. In addition, the EWG began to push the Food and Drug Administration in 2007 to update and improve cosmetic product regulations by urging the agency to set stricter standards to better protect public health [14].

Due to the ecotoxicological risks of organic UVFs, using inorganic UVFs for replacement has become a topic of interest for both producers and consumers. Although organic UVFs have dominated the market for PCPs in the past, inorganic UVFs as substitutions are increasing due to their broad UV spectrum protection and limited skin penetration and health risks [23,24]. It is believed that 60% of nTiO_2_ and 80% of nZnO produced globally are used in cosmetic products [25,26]. With the increasing production and application, the discharge of inorganic UVFs into environments is inevitable. In the United States, hundreds of tons of TiO_2_ and ZnO are disposed of in the environment every year [27]. To date, studies have shown that inorganic UVFs have been detected in marine waters, sediments, and organisms at increasing concentrations [1]. For example, Botta et al. [28] estimated that in reef areas, 36–56 tons of TiO_2_ were released from sunscreens, where the concentration of TiO_2_ could reach tens of milligram liters in surface microlayer [4]. Inorganic UVFs are prone to persisting in the environment due to continuous emissions and refractory degradation, which pose health threats to aquatic organisms at different trophic levels.

We comprehensively reviewed the current study status of the environmental sources, occurrences, behaviors, and impacts of sunscreen-derived inorganic UVFs in aquatic environments. The associated primary nanoparticle characteristics and coating materials significantly affect the environmental behavior and fate of inorganic UVFs. The consequential ecotoxicological risks and underlying mechanisms are discussed at the individual and trophic transfer levels. Accordingly, suggestions are given for future study and recommendations for the scientific attention and control of inorganic UVF-containing products.

## 2. Inorganic UVFs in Aquatic Environments

### 2.1. Sources and Occurrences

**Figure 1:**
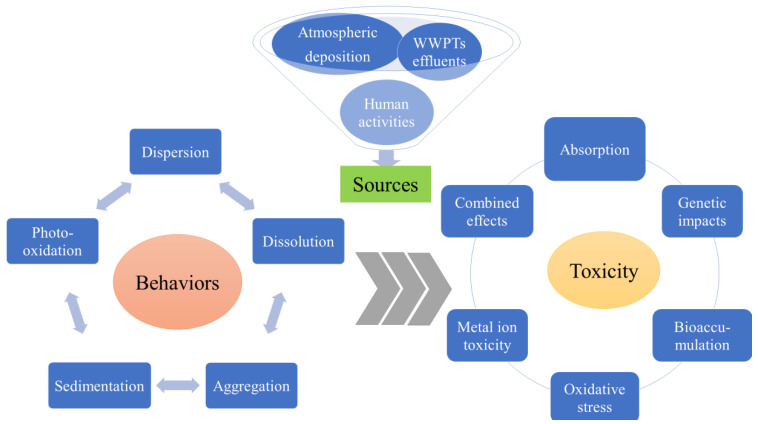
The sources, behaviors, and toxicity of sunscreen-derived inorganic UVFs in aquatic environments.

UVFs have been detected in surface waters [29], urban groundwater [30], sediments [31,32,33], marine water, and biota [1,34]. The environmental sources and distribution of organic UVFs have been well reviewed in recent years [1,34]. However, very little is known about the occurrences and distributions of the two increasingly used inorganic UVFs (nTiO_2_ and nZnO). It has been shown that these substances are released into waters, either directly through human activities or indirectly through wastewater treatment plant (WWTP) drainage and atmospheric deposition (shown in Figure 1) [11,29,35]. Some studies have indicated that there is a direct relationship between the amounts of sunscreen components in waters and recreational activities, such as swimming, diving, surfing, etc [4,36,37]. In addition, the effluents of WWTPs and domestic sewage indirectly release UVFs, as sunscreen components cannot be completely removed [6,11]. Atmospheric aerosols containing UVFs may occur from different sources, including directly after spraying sunscreen on the skin, with effluents from WWTPs, and indirectly with the incineration of WWTP sludge.

According to a survey study, there are approximately 16,000–25,000 tons per annual (t/a) of sunscreens that contain nTiO_2_ in tropical countries, and at least 25% of sunscreen applied to the skin enters the ocean during water recreational activities [9]. It is estimated that the content of nTiO_2_ in sunscreens is approximately 4%, and the amount of nTiO_2_ released annually is approximately 160–250 t in these tropical countries [1,38]. Specifically, Sánchez-Quiles and Tovar-Sánchez [9] estimated that over 4 kg of nTiO_2_ can be released from sunscreen into seawater during a summer day on a tropical touristic beach. Another study suggested that the recreational activities that take place at Old Danube Lake (Vienna, Austria) may involve the consumption of sunscreen of 8.1 t per year, and they estimated that 94.5 kg of TiO_2_ per year may be released into lake waters [39]. A recent study has shown that inorganic UVFs present in the formulation of sunscreens are detected in nearshore water and are concentrated in the surface microlayer that ranges from 6.9 to 37.6 mg/L for TiO_2_ and from 1.0 to 3.3 mg/L for ZnO [4].

### 2.2. Environmental Behaviors

The specific behavior of inorganic UVFs released from sunscreens into aquatic environments has not been well addressed. As sunscreen is a complex chemical mixture; once it is in water, the inorganic UVFs released from sunscreen are complex and can exist in the form of aggregates of various complex components [40,41], including surface-modified complexes or raw NPs. For raw NPs, their environmental fate generally includes dispersing, aggregating, and dissolving/releasing metal ions and settling onto sediments or being absorbed and bioaccumulated by organisms (shown in Figure 1) [28,39,42]. Many studies have confirmed that nZnO UVFs rapidly dissolve in water and form hydrated Zn^2+^ cations [43,44]. Other inorganic UVFs, e.g., nTiO_2_, are regarded as relatively stable and rather insoluble in water [45]. Thus, these UVFs tend to aggregate into larger particles, which remain suspended or precipitate to the bottom of the aquatic environment. In general, the higher the content of UVFs, the higher the SPR the sunscreen obtained. For organic UVFs, they absorb UVR, thus their spectral characteristics determined the absorbance of UVR as well as the sun protection factor (SPR); most of them are photo-instability effects related to UVR exposure [46]. For inorganic UVFs, they mean to scatter and reflect UVR; thus, they are more stable than organic UVFs, but their particle size would affect the SPR and transparency (aesthetics of the products), thus most inorganic UVFs are nanosized. The stability of physical sunscreens was influenced by the coating materials, with these organic materials in physical sunscreens tend to perform photodegradation and photo-instability effects related to UVR exposure, thus making inorganic UVFs easier to bear in the environment [46]. In addition, photooxidation and photodegradation are also proposed to occur when inorganic UVFs are exposed to sunlight. Inorganic UVFs, including nTiO_2_ and nZnO, are often used as photocatalytic materials; once released into water, they can be photooxidized during irradiation by ultraviolet light and generate hole-electron pairs; reactive oxygen species (ROS) are produced when hole-electron pairs react with H_2_O or O_2_ on the surface of NPs, which also decreases the particle size and produces more ROS [47,48]. Studies have shown that inorganic UVFs are photooxidized, produce ROS, and cause photocatalytic toxicity to aquatic organisms [49]. In addition to these behaviors, inorganic UVFs easily settle into sediments due to gravitational force, thereby aggregating into larger NPs. UVFs, both the organic and inorganic varieties, are absorbed or captured by aquatic organisms during the above processes, which causes damage to organisms and even bioaccumulation in organisms or sediments in the water. We recently found that physical sunscreens and related inorganic UVFs exhibit bioattachment on the surfaces of button coral and cause significant growth inhibition and expulsion of zooxanthellae (*Symbiodinium* sp., unpublished data), which demonstrates the importance of further exploring the environmental fate of inorganic UVF-containing cosmetic products and the derived UVFs.

The nTiO_2_ and nZnO were dispersed (partial dissolved) in physical sunscreens during the manufacturing process, which would be modified first sometimes. Thus inorganic UVFs in sunscreens often exist as surface-modified complexes. For surface-modified complexes, their potential environmental behavior presents some differences that need to be discussed. Primarily, coexisting surface coatings affect the fate of NPs to some extent. In addition to UVFs, sunscreens also contain other ingredients, such as preservatives (e.g., paraben derivates) [50], coloring agents (e.g., ammonium sulfate, ferric ammonium ferrocyanide, copper powder, and iron) [51], film-forming agents (e.g., acrylates and acrylamides) [52], surfactants, chelators, viscosity controllers (e.g., potassium cetyl phosphate and pentasodium ethylenediamine tetramethylene phosphonate), and fragrances [53]. Some of these ingredients have been detected in coastal waters [54,55,56]. Thus, nTiO_2_ (and nZnO) may be present in the form of bare or coated NPs in the aquatic environment, and their dimension, shape, crystal phase, and surface area vary among different sunscreen products [27]. A recent study showed that sunscreen-derived nTiO_2_ exhibits a larger particle size but a smaller hydrodynamic diameter and lower zeta potential than industrial uncoated nTiO_2_, which exhibits significant aggregation [57]. In contrast, the presence of carboxymethyl cellulose (CMC) or polyvinylpyrrolidone (PVP) significantly enhances the stability of uncoated nTiO_2_, as determined by the zeta potential values measured at pH 7, with substantial shape changes that result in spherical particles and relatively small nTiO_2_ sizes [57]. Similar substantial shape transformations induced by stabilizers have been found in other studies [58,59]. Inorganic UVFs generally have a small particle size, strong hydrophobicity, and are insoluble in water; thus, Brownian motion, eddy motion, and runoff shear force result in some inorganic UVF particles remaining in suspension [60]. Engineered polymers or organic and inorganic substances that serve as coating materials or act as stabilizers have been found to modify the physicochemical properties of raw NPs, thereby affecting particle stability and mobility through electrostatic repulsion [61,62,63] and by maintaining the dispersion of nanosized inorganic UVFs. For example, nTiO_2_ has been found to be fully dispersed and stabilized in natural water that contains organic materials [64]. Therefore, the stability of inorganic UVFs depends on their physicochemical properties and coating materials [27,57].

An early study indicated that eight of nine commercial sunscreen products are coated with nonvolatile inorganic residues, typically Al_2_O_3_ or SiO_2_, to minimize the photochemical activity of TiO_2_ [27]. Adsorbed or covalently bonded surfactants affect aggregation stability by increasing the surface charge and electrostatic repulsion or by reducing the interfacial energy between the particles and the solvent [65]. The interaction between steric repulsion and universal Coulomb attraction is caused by the surface coating layers, which may profoundly affect the aggregation kinetics. However, a recent study showed that sodium citrate provides higher stability for spherical nTiO_2_ than PVP, sodium dodecyl sulfate, and polyethylene glycol, since sodium citrate results in lower critical coagulation concentrations [66]. Additionally, another study showed that the addition of coating materials such as CMC, PVP, and silica prevents significant TiO_2_ aggregation by facilitating dispersion [60]. These stabilizers change the physicochemical properties (particle sizes and zeta potential) of nTiO_2_ and produce a stable TiO_2_ suspension with a cluster size smaller than that of uncoated nTiO_2_ because they play the role of a dispersant that prevents nanoparticle aggregation [57]. A decrease in particle size results in a higher proportion of atoms on the particle surface, which alters the electronic structure, surface charge, and final degree of aggregation [67]. Small particles with high surface energy aggregate more readily than larger particles since aggregation reduces the free energy in the NP system.

It has been revealed that the dissolution of inorganic UVFs depends on the solubility of the materials themselves and on the concentration gradient in water [68,69]. For example, nZnO releases more Zn ions in seawater with a higher ionic strength than in fresh water [70]. Moreover, the dissolution of inorganic UVFs is clearly affected by the physicochemical properties of the material, such as the particle size, shape, and surface coating. Generally, the solubility of NPs is higher than that of the bulk phase because the decreased size increases the specific surface areas and the enthalpies of the formation of the ions [71]. Fairbairn et al. [72] also pointed out that nZnO is more easily dissolved in sea water than ZnO with ordinary particle sizes or Fe-doped nZnO. However, for nZnO, the impact of different sizes on dissolution is not as obvious for nanosized, bulk, or large particles due to the high solubility of ZnO, which can exhibit up to 80% dissolution [69,73,74]. Additionally, the shapes of NPs have been shown to affect both the rates of dissolution and the equilibrium concentrations [14]. The dissolution rate for spherical nCuO is faster than that of rod and spindle nCuO [75], while spherical nZnO induces lower toxicity than rod-shaped nZnO because the actual Zn ion concentration that results from the dissolution of rod-shaped nZnO is much higher than that of spherical nZnO [76].

Quite often, the dissolution rate of inorganic UVFs significantly decreases in the presence of surface coatings because the surface coating acts as a physical barrier or shield that prevents electrons or photons from reaching the NP surface [77]. In sunscreens, photoactivity problems may arise if particles are not treated with coatings, and manufacturers commonly employ inert surface coatings that dramatically reduce the potential for photoactivity; existing data suggests that these surface coatings reduce UV reactivity by as much as 99% [40,41]. However, organic coatings slow the dissolution process relative to that of uncoated ZnO but lead to an increased concentration of Zn^2+^ at equilibrium [78]. Otherwise, if the coatings are not stable or if manufacturers use forms of ZnO or TiO_2_ that are not optimized for stability and sun protection, then sunscreens may not be protective [14]. These results suggest that inorganic UVFs might input substantial amounts of free metals into an aquatic environment and pose a toxicity risk to aquatic ecosystems.

In addition to the influence of internal NP properties, external environmental factors such as light, pH, and natural organic matter (NOM) can also make a difference. The interaction energy barrier decreases with a decreasing particle size according to the Derjaguin–Landau–Verwey–Overbeek (DLVO) theory, and it is affected by the properties of the primary NPs (e.g., size, shape, chemical composition, and surface coatings), solution chemistries (e.g., pH, ionic identity, electrolyte patterns, and reactions with NOM), and environmental conditions (e.g., temperature and dissolved oxygen level) [69,79]. For example, a large proportion of nZnO dissolves at a limit close to the solubility limit of ZnO(s) at a high pH of approximately 8.2, and both visible and UV light facilitate nZnO dissolution at lower pH values that range from 4.8 to 6.5 [80]. Light warms the water, enhances the release rates of inorganic UVFs, shortens the equilibrium time and even increases equilibrium concentrations [62]. Moreover, inorganic UVFs generate ROS under irradiation with visible and UV light; this results in the oxidation of metal ions and surface organic compounds, which increases the dissolution rates due to the decomposition of surface coatings and loss of the stabilizing effect of dissolved organic matter. The influence of solution properties on the dissolution of inorganic UVFs is dynamic and complex [62].

### 2.3. Substantial Environmental Impacts

The discharge of inorganic UVFs from sunscreens into waters is concomitant with the input of several other constituents, including nutrients (e.g., silicates, phosphates, and nitrates), metals (e.g., Al, Cd, Cu, Co, Mn, Mo, Ni, Pb, and Ti), and coating materials (e.g., preservatives, coloring agents, film-forming agents, surfactants, and stabilizers). Many of these coexisting substances are persistent; therefore, their effects might last beyond the most recent period of sunscreen use. These additional constituents influence the bioavailability and degradability of sunscreen ingredients since the biogeochemical routes into environmental media (water, sediment, and biota) and the hydrophobicity or hydrophilicity of the substances contained in sunscreens are diverse and complex [1,81]. Moreover, the effects of sunscreen contamination (especially from commercial formulations instead of individual compounds or ingredients) are sometimes difficult to perceive in laboratory studies because of their complex matrix [82,83] and unknown composition [84]. Additionally, because of the diverse formats of sunscreens (e.g., cream, gel, spray, and oil), their dilution and release of UVFs into water are different, as are their bioavailabilities and toxicities [4,85].

It is likely that environmental exposure to inorganic UVFs and the chemicals contained therein results from the production and consumption of sunscreens. Studies have indicated that UVFs and other ingredients from sunscreens have been detected in the tissues of marine organisms, such as clams, oysters, gastropods, and fish [86,87], and have shown toxicity in some aquatic species, such as the crustacean *Daphnia pulex* and the fish *Danio rerio* [88,89]. Rodríguez-Romero et al. [90] demonstrated with laboratory experiments and field measurements that sunscreens are an important source of nutrients, such as nitrogen compounds (NO_3_^−^, NO_2_^−^, and NH_4_^+^) and phosphate (PO_4_^3−^) in coastal marine environments, raising the possibility of algal blooms in oligotrophic waters. More specifically, some concentrations of the compounds (e.g., those of PO_4_^3-^, NH_4_^+^, NO_3_^−^, and Ti) released into water vary during the course of a day, which is known to be associated with variations in beach-goer activities and changes in solar radiation [4]. Sunscreens have also been identified as sources of high-risk metal substances [91], many of which (e.g., Al, Zn, Mg, Fe, Mn, Cu, Cr, and Pb) have been detected and quantified in aquatic environments [4,92]. Moreover, the organic components of sunscreens are readily removed from particle surfaces [93,94], which leaves the inorganic UVFs exposed to the surrounding environment. Although the ecological relevance of this input has not been well reviewed, Tovar-Sánchez et al. [4] suggested that it could enhance primary production in the oligotrophic waters of the Mediterranean Sea.

In addition to the direct output of soluble substances from sunscreens, some indirect metabolites are also produced in the water environment under sunlight. A study carried out on a touristic beach indicated that both temporal (daily) and vertical (water column) distributions of H_2_O_2_ concentrations generated by inorganic UVFs (nTiO_2_ and nZnO) were present in marine waters [9]. According to the authors, the concentrations of H_2_O_2_ found within the top centimeter of the surface layer were up to 41.6% higher than those in the immediate subsurface waters [9]. Similarly, a large number of studies have indicated that nTiO_2_ and nZnO produce ROS under sunlight exposure and induce oxidative stress in organisms [62,95,96,97,98]. Therefore, more reliable information is required on the role of sunlight in the release of the main ingredients and byproducts of sunscreens into water.

Accordingly, sunscreen-derived inorganic UVFs are very likely to be released into the main water bodies of lakes, rivers, and oceans but do not remain suspended for a long time, with the most likely fates being aggregation, dissolution, and settling onto the sediments due to the water chemistry conditions and the presence of natural colloids. However, their environmental behaviors will be affected by the surface coating and various physical and chemical factors, such as ocean currents, waves, and high salinity, and they will undergo complex aggregation and dissolution reactions; moreover, their structural form, distribution, and toxic effects will constantly change. Nevertheless, these behaviors and transformation processes for inorganic UVFs must influence their bioavailability and toxicity, which cause great impacts on natural aquatic ecosystems [80].

## 3. Toxicity of Inorganic UVFs on Aquatic Organisms

The adverse effects of organic UVFs on aquatic organisms have been reviewed in recent literature [21], but studies on the ecological risk of inorganic UVFs are limited. Although studies have found that inorganic UVFs do not cause more damage to humans than organic UVFs [34,99,100,101], notably, the potential environmental effects of UVFs on aquatic organisms are not taken into consideration during their production, and even worse, few specific recommendations for the environmentally friendly use of sunscreens have been offered by agencies or governments worldwide.

### 3.1. Interaction of Inorganic UVFs with Organisms in Aquatic Environments

Although inorganic UVFs are often coated with complex stabilizers, they are released in particle form when sunscreen enters the water. When they enter the water environment, inorganic UVFs tend to disperse, aggregate, dissolve metal ions, settle, absorb, and/or bioaccumulate within organisms. Studies have shown that inorganic UVFs interact with aquatic organisms in a variety of ways [83,102]. First, inorganic UVFs or their aggregates can adsorb or wrap themselves around the surface of phytoplankton or microorganisms and eventually be ingested by organisms. Second, filter-feeding or devouring animals, such as planktonic amphipods, benthic shellfish, and polychaetes, can filter or swallow inorganic UVFs directly. Third, organisms of high trophic levels can directly consume water that contains inorganic UVFs or algae and other low trophic level organisms, and thus cause the accumulation, transfer, and even magnification of inorganic UVFs along the food web and result in unpredictable environmental effects and ecological risks.

### 3.2. Toxicity of Inorganic UVFs on Organisms at the Individual Level

Sunscreen-derived inorganic UVFs are widely distributed in all levels of water, including the surface microlayer, water column, and sediment, which also results in interactions with various environmental factors; thus, they are deemed to cause adverse effects on various organisms in the aquatic environment. It is still difficult to conduct exposure experiments specifically for sunscreen-derived inorganic UVFs since sunscreens in water release not only inorganic UVFs but also many latent toxic chemicals, such as surfactants. Thus, there is little direct laboratory evidence of the damage caused by sunscreen-derived inorganic UVFs that primarily focuses on nTiO_2_ UVFs and nZnO UVFs in aquatic organisms (Table 1).

#### 3.2.1. nTiO_2_ UVFs

Only a few studies have focused on the toxicity of inorganic UVFs to marine algae. Early findings suggested that the nTiO_2_ from sunscreens alters the species density and composition of the microalgae community due to the impairment of cell growth; sunscreen toxicity levels are significantly related to UVR, which is commonly neglected in some bioassays, but this could alter the results in important ways and should be considered when performing environmentally relevant bioassays [104]. Because of its photochemical properties, nTiO_2_ produces high concentrations of H_2_O_2_ as a result of UVR [9], which causes toxic effects such as damage to cell membranes or cell walls [93], lipid peroxidation, growth inhibition, and a decline in the proportion of healthy cells in microalgae populations [107]. Furthermore, the adsorption of nTiO_2_ particles on the surfaces of algae cells can cause physical damage, such as shading effects, which inhibit cell growth [108].

Direct toxicology data on the effects of sunscreen-derived inorganic nTiO_2_ on zooplankton, fish, and benthos are rare [49,103,104,105,106]. A recent study indicated that nTiO_2_ released from sunscreens causes repellency and mortality in shrimp (*Palaemon varians*) and speculated that the avoidance response might be the main factor responsible for the reduction in the shrimp population due to increasing sunscreen concentrations at the local scale [85]. In addition, the nTiO_2_ released from sunscreens impairs sea urchin development or causes malformations due to a decrease in AChE activity [49,105]. In realistic environmental scenarios, the self-aggregation of inorganic UVFs into larger masses and their incorporation into aggregate materials might increase the bioavailability and toxicity for algae, phytoplankton, zooplankton, and benthos along food chains.

#### 3.2.2. nZnO UVFs

nZnO can absorb ultraviolet A-rays (UVA) and ultraviolet B-rays (UVB), while nTiO_2_ can only absorb UVB; therefore, nZnO provides better UV protection than nTiO_2,_ and its use in physical sunscreens may even exceed that of nTiO_2_ in the future [109]. Few studies have assessed the potential release and toxicity of sunscreen-derived nZnO in aquatic environments [43,49,85]. For instance, studies conducted with zooplankton and benthic animals exposed to nZnO-containing sunscreen showed repellency and mortality effects in shrimp [85], irreversible coral bleaching, and widespread mortality of symbiotic zooxanthellae [43], which primarily resulted from Zn^2+^ toxicity. Moreover, studies have shown that the toxicity of nZnO UVFs appears to be related to solubility or the release of toxic metal ions (Zn^2+^) instead of aggregation, which leads to the conclusion that higher Zn^2+^ solubility is accompanied by higher toxicity [110]. Similarly, the nZnO released from sunscreens has caused impairments or malformations in sea urchin development due to Zn^2+^ internalization [49,105]. These results indicate that the solubility of nZnO plays a critical role in the toxicity of physical sunscreens to marine organisms [11].

It has been reported that the surface properties of inorganic UVFs, including the pH and ionic strength of the solution, affect their solubility, which largely determines the extent of toxicity [111,112]. Attempts have been made to reduce solubility and, consequently, ZnO toxicity through iron doping. Although this strategy has been shown to reduce ZnO cytotoxicity in cell cultures [113], Fairbairn et al. [72] found that 10% iron-doped ZnO is just as toxic as non-doped ZnO to sensitive marine embryos. The solution pH and ionic strength may affect the adsorption of NPs onto cells due to changes in surface charges [114,115,116]. In addition, Peng et al. [117] reported different sensitivities to nZnO in three marine diatoms (*Thalassiosira pseudonana*, *Chaetoceros gracilis*, and *Phaeodactylum tricornutum*) and introduced the idea that the morphologies of nZnO samples also affect their toxicities. These results confirm that the toxic mechanisms of inorganic UVFs are related to various toxic factors; thus, more systematic studies are needed to elucidate their toxicity profiles.

### 3.3. Impacts of Inorganic UVFs on Multiple Trophic Levels

Given the persistence and stability of inorganic UVFs such as nTiO_2_, organisms can accumulate and even transfer these substances along food chains [35,49,85,118,119]. Previous studies have shown that nTiO_2_ and nZnO can be internalized into the cells of bacteria and algae and accumulate in aquatic organisms, including zooplankton, swimming organisms, and benthos [1,83,120,121]. Notably, it is highly possible that inorganic UVFs are transferred from lower trophic organisms to higher trophic organisms through predator-prey relations and biomagnification in the food web [122,123]. In fact, the bioaccumulation of chemicals released from sunscreens has been detected in fish and mussels [124,125,126], while the mechanisms by which inorganic UVFs transfer in a food web are still not clear. Studies have shown significant amounts of nTiO_2_ in the dietary exposure groups, which indicates that dietary intake may constitute a major route of trophic transfer [123]. For nZnO, the transfer behaviors can be divided into particle and metal ion accumulation routes since nZnO easily dissolves to produce Zn^2+^. Considering that some aquatic organisms, such as fish and clams, are human food sources and provide food for wildlife, the bioaccumulation and trophic transfer of inorganic UVFs along the food chain have raised increasing concerns.

### 3.4. Potential Mechanisms for the Toxicity of Sunscreen-Derived Inorganic UVFs

Since the two most commonly used inorganic UVFs, i.e., nZnO and nTiO_2,_ are NPs, they share similar behaviors in aquatic environments, as mentioned above. Therefore, it has been hypothesized that the toxicity of sunscreen-derived NPs might arise from mechanisms similar to those of raw nTiO_2_ and nZnO. Although the aquatic toxicities of raw nTiO_2_ and nZnO have been well-studied in previous reviews [22,60,127], the toxicological evaluation of the mechanism on sunscreen-derived inorganic UVFs with aquatic organisms has only recently begun, and few studies have assessed the toxic performance of sunscreen-derived NPs compared with those of engineered raw NPs [106].

**Figure 2:**
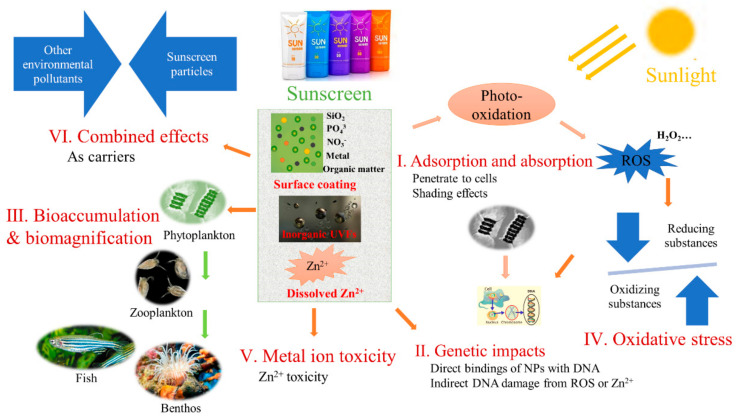
Potential mechanisms for sunscreen-derived inorganic UVF toxicity in aquatic organisms.

As shown in Figure 2, adsorption or absorption is important and constitutes the first step in the interaction between NPs and aquatic organisms. Engineered raw NPs may attach to the surfaces of aquatic organisms and cause physical effects such as shade photosynthesis, direct mechanical damage to phytoplankton, or blocking vital movement in zooplankton [60]. Wang et al. [128] reported that nTiO_2_ significantly inhibits *Phaeodactylum tricornutum* growth directly through physical effects such as cell wall damage that arises from algae entrapment. Although we recently found that sunscreen-derived inorganic UVF particles can be absorbed on the surfaces of button corals (unpublished data) and result in the contraction of tentacles, related reports are rare; thus, more studies are encouraged with other aquatic organisms to provide direct evidence.

Internalization has been deemed a common pathway for the uptake of engineered NPs by algae [128,129]. Once they penetrate the cell barrier, NPs can undergo translocation into the intracellular environment via diffusion or endocytosis [130]. Here they can interact with DNA or attach to organelles in cells and block normal function or cause genetic impacts [60,127]. Genetic effects may be produced by the direct binding of NPs with DNA, by the indirect damage from the ROS generated by NPs, or by the toxic ions released from soluble NPs [60]. Although few studies have directly demonstrated the genetic damage induced by sunscreen-derived NPs, the ROS generation or Zn^2+^ dissolution from physical sunscreens can impact the DNA or RNA of aquatic organisms. In particular, small single NPs (<10 nm) can reach the nucleus through nuclear pores, while larger NPs may also have the opportunity to bind with DNA molecules when the nuclear membrane dissolves due to the division of cells during mitosis. The overall uptake of the NPs that reach the nucleus through diffusion across the nuclear membrane or that are transported through nuclear pore complexes presents the danger of subsequent direct interactions with cellular genetic material [60].

Following attachment, NPs may accumulate on cell surfaces or transfer to specific organs or tissues (e.g., stomach, gills, and liver) for storage [60]. Previous studies have shown that metal-based NPs can be ingested and can accumulate in single aquatic organisms [60,131] or undergo trophic transfer in the food chain [122,123], especially with higher trophic level organisms such as fish or filter-feeder organisms such as fleas and many benthic organisms (e.g., mussels, oysters, and clams), after waterborne or foodborne exposure [117,132,133,134]. The bioaccumulation of nTiO_2_ and nZnO has been shown to inhibit the growth of aquatic organisms [117,132]. In fact, there is evidence that bioaccumulation is directly related to the toxicity of NPs [135,136]. These studies show that NPs mainly accumulate in specific organs or tissues in aquatic organisms and thus inhibit their biological intake and affect their biological metabolism and energy acquisition. Notably, NP absorption and bioaccumulation cause physical damage and then lead to adverse consequences for organisms, including oxidative stress, behavioral inhibition, and death. However, studies on the bioaccumulation of sunscreen particles are scarce. Although we recently found that the active components of physical sunscreen (Ti and Zn) are bioaccumulated in button corals (unpublished data), we still have not clarified how they enter coral individuals, the organs or tissues in which they prefer to accumulate, or the consequences that ultimately result. A remaining question is whether sunscreen-derived NPs exhibit toxic mechanisms similar to or different from those of raw NPs, since limited studies have only recently been published (shown in Table 1). However, the availability of studies on raw engineered NPs definitely shows that further studies to elucidate the toxicity profiles of physical sunscreens are urgently needed.

The specific toxicity of the MoA to metal-based NPs is related to ROS generation and subsequent ROS-induced oxidative stress. Oxidative stress and cellular toxicity are of concern because nZnO and nTiO_2_ can penetrate the stratum corneum, enter the dermis, and ultimately reach the blood supply [120,137,138,139,140]. Previous results have suggested that the physical interactions of NPs induce significant oxidative stress, which provides direct evidence for the toxicological impact of engineered raw NPs in aquatic organisms [128,135]. In general, both sunscreen-derived and raw NPs can undergo photooxidation and generate ROS under sunlight irradiation, and ROS overgeneration is deemed to result in subsequent cell membrane damage, lipid peroxidation, growth inhibition, and other negative impacts [103,141,142]. Sunscreen-derived nTiO_2_ has been indicated to induce the photocatalytic generation of ROS, such as H_2_O_2_, in vitro and cause growth inhibition and distribution changes in algae [9,103,104,143]. Moreover, inorganic UVFs enter aquatic organisms and induce ROS generation in vivo, which causes toxicological impacts on *Chlorella* spp. [144]. The production of ROS, either in vitro or in vivo, directly or indirectly, causes oxidative stress. According to a study on raw NPs, the in vitro aqueous production of ROS by raw NPs requires photosensitization; that is, the production of ROS is driven by light, especially UVR. Although the generation of ROS is instantaneous, ROS are usually quenched within seconds by reducing substances, and it has been reported that ROS produced in vitro exert harmful effects on organisms [9,60,104,145]. However, most related studies have been conducted to probe ROS generation in vivo and provide direct evidence of oxidative stress after NP exposure [128,135]. Unlike raw engineered NPs, sunscreen-derived NPs are often coated or modified when they are applied to cosmetic products. Once sunscreen is in water, inorganic UVFs released from sunscreen are complex and can exist in the form of aggregates of various complex components [40,41], including surface-modified complexes or raw NPs. To our knowledge, no study has been conducted to clarify whether coating modifications alter photosensitivity or affect the extent or duration of oxidative stress. However, for sunscreen-derived nTiO_2_, studies have indicated that their toxicity is also affected by coexisting coatings, which might have determined their aggregated sizes or the levels of ROS generated. Studies have shown that UVFs coated with inert protective films (such as SiO_2_, Al_2_O_3_, or organic matter) or coating materials such as organomodified silicon oxide exhibit significantly reduced production of ROS on the surfaces of NPs and alter the impacts of ROS on organisms, even during UVR [146,147]. That is, coating materials alleviate the impacts of ROS that result from sunscreen-derived UVFs on organisms. It is easy to understand that the coatings and modifications are meant not only to shield or reduce UV damage but also to prevent the adverse biological effects of UVFs [147]. Oxidative stress should be a common toxicity MoA for the two types of particles, but differences exist in the detailed MoA and sites affected (in vitro or in vivo). Raw engineered NPs are more often focused on the generation of ROS in vivo, while sunscreen-derived NPs are more often focused on the generation of ROS in vitro. Since these coating materials affect the behavior and toxicity of sunscreen-derived inorganic UVFs, the impacts of the different coating materials and their diverse characteristics on the toxicity of inorganic UVFs should be given more attention and considered during the development of safe sunscreens.

Furthermore, as with oxidative stress, metal ions can be released from both sunscreen-derived and raw NPs, which plays an important role in their toxicity to aquatic organisms. In contrast to the largely insoluble nTiO_2_, nZnO can rapidly dissolve as Zn^2+^ in water [44], and Zn^2+^ is the major contributor to the toxicity of sunscreen-derived nZnO [49]. For example, nZnO toxicity to the marine diatom *Thalassiosira pseudonana* has been solely explained by the Zn^2+^ reaction [148]. Zn^2+^ toxicity constitutes another unique toxic MoA for nZnO UVFs. For raw engineered nZnO, the toxicity can be ascribed to Zn^2+^ concentrations; however, the coatings of sunscreen-derived nZnO often delay the dissolution equilibrium and lead to an increased concentration of Zn^2+^ cations at equilibrium [78]. Spisni et al. [106] reported that the toxicity of sunscreen-derived nZnO for the growth of algae (*Thalassiosira pseudonana*) appears to be lower than that of raw nZnO at relatively low concentrations, but the toxicity levels become similar when concentrations are increased to 50 mg/L. Recently, Corinaldesi et al. [43] found that sunscreen-derived nZnO induces the complete and latent irreversible bleaching of stony coral and rapid and widespread mortality of symbiotic zooxanthellae. Presumably, these effects are attributable to the toxicity of Zn^2+^, which causes alterations in the composition of the cellular membrane lipids of hard corals and their symbiotic organisms [149]. This is of concern because an increasing number of manufacturers are using ZnO rather than TiO_2_ in sunscreens.

Accordingly, the MoAs for the toxicity of sunscreen-derived inorganic UVFs are similar to those of raw engineered NPs, but they exhibit some differences due to the complex surface coatings and modifications. Sunscreen-derived NPs exist in forms that are more complicated than those of raw NPs, and nanoparticle monomer toxicity, agglomeration toxicity, or complex mixed toxicity may result when they enter water. In contrast to engineered NPs, inorganic UVFs are often coated with stabilizers in sunscreens to prevent aggregation [27,93,150,151]; thus, they result in altered interactions with organisms [152] and differences in the extent of toxicity [104,106]. Compared with raw nTiO_2_, the presence of some stabilizers increases the toxicity of NPs and the inhibition of growth in *Escherichia coli* (*E. coli*) [57]. Moreover, the sizes of TiO_2_ particles are relatively small and appear to contribute to *E. coli* cell damage [60], and nTiO_2_ samples with small particle sizes, large surface areas, and strong electrostatic attractions easily act as carriers of other environmental pollutants [136,153,154], including the other components of sunscreens, which affect their toxicity to aquatic organisms. Accordingly, although sunscreen-derived inorganic UVF-engineered NPs exhibit some similarities in toxicity and MoA, the presence of surface coatings or modifications is known to cause differences and result in different toxicities; thus, further study is required to increase our understanding of these differences and their origins.

## 4. Conclusions and Future Perspectives

This study reviewed the fate and toxicity of inorganic UVFs in aquatic environments, with information on their sources, environmental behaviors, and toxicities to aquatic organisms from the individual to the trophic transfer levels. Inorganic UVFs derived from sunscreens are often dispersed, aggregated, dissolved into waters, and settled into sediments, and they tend to be absorbed and bioaccumulated by organisms; this results in adverse effects on various organisms in the aquatic environment, which are directly influenced by various environmental factors and the presence of coatings; resulting in different environmental fates and toxicities compared with raw engineered NPs.

Inorganic UVF-containing sunscreens are deemed to be a source of multiple environmental pollutants, and they pose new environmental risks to aquatic environments. As indicated by data on coastal-zone population growth and tourism activities, sunscreens exhibit the fastest growth in global sales. This fact, together with recent research that indicates the presence and accumulation of UVFs in environmental media, emphasizes the potential damage that could be caused in marine areas. Thus, future investigations are needed to understand the magnitude and real impacts of these emerging pollutants in marine systems, including studies on the distribution and partitioning in the water column, dissolution and speciation of their main components, evaluation of the ecological significance of nutrient input, and residence time, aging, persistence, accumulation, and toxicity in the trophic chain.

Most studies on the environmental behaviors of inorganic UVFs have been conducted under laboratory conditions, which may not represent realistic natural environments. Although some recent studies have investigated the aggregation, dissolution, and transformation of UVFs in natural water bodies by collecting lake water and seawater samples, knowledge of the environmental fate of inorganic UVFs in the real environment is still limited. In fact, UVFs can be greatly impacted by various factors in the natural environment, which complicates their behavior. Thus, further studies should be conducted under realistic environmental conditions to the fullest extent possible.

Moreover, a thorough understanding of the causal relationship between the properties of inorganic UVFs and toxicity remains largely elusive. Although many studies have been performed on the implications of these NPs for aquatic organisms, there is an insufficient characterization of the material properties and the relationship between the observed toxicity and specific features of inorganic UVFs, such as Zn^2+^ toxicity, bioaccumulation, shading effects, and ROS generation. Thus, establishing a quantitative correlation between environmental behaviors and toxicity would facilitate the future evaluation and prediction of the toxicity of related cosmetic products.

Finally, many previous studies have attributed the toxicity of inorganic UVFs to one or two major aspects of material properties or solution behaviors. Nevertheless, material properties are often interrelated and interdependent. Moreover, after undergoing the abovementioned processes, the coating materials, size distribution, and surface properties of the particles will be dramatically affected. Thus, tracking dynamic aggregation or disaggregation to determine the actual fractions of nanosized inorganic UVFs and aggregated or agglomerated particles at cellular interfaces remains the most important issue identified thus far.

Notably, regardless of the recommended usage level and the ways in which people use cosmetic products, the potential environmental effects of UVFs on nontarget organisms were not taken into consideration when governments and agencies developed their recommendations. In particular, although large quantities of sunscreen can be released directly into seawater during recreational activities carried out on hot days, there are very few specific recommendations for the use of sunscreens in coastal areas. Therefore, the ecotoxicological testing of whole products should be included in future assessments of environmental risks and in developing recommendations and regulations for the usage and formulation of commercial sunscreens.

## Figures and Tables

**Table 1:** Toxicity and potentially toxic mode of action (MoA) of inorganic UV filters on aquatic organisms.

Inorganic UVFs	Organism	Exposure Conditions	Effects	MoA	References
TiO_2_ (release from cosmetic products)	Algae (*Thalassiosira pseudonana*)	0–96 h; 0.13–100 mg/L	Growth inhibition	Potential ROS production	[103]
nTiO_2_ from sunscreens	Chaetoceros gracilis (*Bacillariophyceae*); Amphidinium carterae (*Dinophyceae*); Pleurochrysis roscoffensis (*Primnesiophycae*); Nannochloropsis gaditana (*Eustigmatophyceae*)	75 h; sunscreens (1–200 mg/L) or nTiO_2_ (1–10 mg/L)	Distribution of phytoplankton	H_2_O_2_ produced adsorption and absorption by the phytoplankton, membrane damage, ROS, and perhaps genotoxic damage	[104]
nTiO_2_ from sunscreen	Sea urchin (*Paracentrotus lividus*)	3 h, 24 h; 10, 20, and 50 μL/L sunscreen	Sea urchin development impairment	decrease in AChE activity	[105]
nZnO (sunscreen-derived)	Algae (*Thalassiosira pseudonana*)	0–96 h, 10 and 50 mg/L	Growth inhibition	Time- and concentration-dependent bioaccumulation	[106]
ZnO from sunscreen	Stony corals (*Acropora* spp.)	48 h of in situ condition 6.3 mg/L	Coral bleaching; release of zooxanthellae	dissolved Zn^2+^ Zn^2+^ shading effects	[43]
zinc-containing sunscreens	Sea urchin (*Strongylocentrotus purpuratus*) embryos	96 h; 0.01–1 mg/L	Malformations (skeletal abnormality, stage arrest, and axis determination disruption)	Zn^2+^ internalized	[49]
nTiO_2_ and nZnO from sunscreen	Shrimp (*Palaemon varians*)	4 h 0–300 mg/L sunscreen	Repellency and mortality effects		[85]

## Data Availability

The data presented in this study are available on request from the corresponding author.
